# Hammett correlation in competition experiments in dissociation of ionised substituted benzophenones and dibenzylideneacetones

**DOI:** 10.1177/14690667231184363

**Published:** 2023-07-05

**Authors:** Nathan W Fenwick, Richard Telford, William H C Martin, Richard D Bowen

**Affiliations:** School of Chemistry and Biosciences, Faculty of Life Sciences, 1905 University of Bradford, Bradford, UK

**Keywords:** Benzophenones, dibenzylideneacetones, α-cleavage, competition experiments, Hammett relationship

## Abstract

A convenient method of applying competition experiments to devise a Hammett correlation in the dissociation by α-cleavage of 17 ionised 3- and 4-substituted benzophenones, YC_6_H_4_COC_6_H_5_ [Y=F, Cl, Br, CH_3_, CH_3_O, NH_2_, CF_3_, OH, NO_2_, CN and N(CH_3_)_2_] is reported and discussed. The results given by this approach, which rely on the relative abundance of [M-C_6_H_5_]^+^ and [M-C_6_H_4_Y]^+^ ions in the electron ionisation spectra of the substituted benzophenones, are compared with those obtained by previous methods. Various refinements of the method are considered, including reducing the ionising electron energy, making allowance for the relative abundance of ions such as C_6_H_5_^+^ and C_6_H_4_Y^+^, which may be formed to some extent by secondary fragmentation, and using substituent constants other than the standard σ constants. The reaction constant, ρ, of 1.08, which is in good agreement with that deduced previously, is consistent with a considerable reduction in electron density (corresponding to an increase in positive charge) at the carbon of the carbonyl group during fragmentation. This method has been successfully extended to the corresponding cleavage of 12 ionised substituted dibenzylideneacetones, YC_6_H_4_CH=CHCOCH=CHC_6_H_5_ (Y=F, Cl, CH_3_, OCH_3_, CF_3_, and NO_2_), which may fragment to form either a substituted cinnamoyl cation, [YC_6_H_4_CH=CHCO]^+^, or the cinnamoyl cation, [C_6_H_5_CH=CHCO]^+^. The derived ρ value of 0.76 indicates that the substituent, Y, influences the stability of the cinnamoyl cation somewhat less strongly than it does the analogous benzoyl cation.

## Introduction

Constructing and interpreting a Hammett correlation is one of the most powerful ways of probing the mechanism of organic reactions.^1–3^ Early efforts to apply the Hammett equation in the development of a rigorous description of the physical organic chemistry that occurs when ions fragment in a mass spectrometer included interpreting appearance energies (then often called ‘appearance potentials’)^4–7^ and the dissociation of ionised acylbenzenes and related species.^8–11^ However, relatively little use of this approach has been made in recent years, perhaps because acquiring the relevant thermodynamic or kinetic data is not always easily achieved by mass spectrometry.

The discovery that many reactions, including condensations, occur at greatly accelerated rates and that new and unusual processes occur in the microdroplets produced by a nebuliser opened new avenues for the application of techniques and equipment routinely employed in mass spectrometry in synthetic and mechanistic organic chemistry.^12–16^ Insight into one such reaction,^17^ the formation of diarylquinoxalines, in microdroplets was obtained by intermolecular competition experiments in which phenylenediamine condensed with either benzil or a 3,3′- or 4,4′-disubstituted benzil and the relative abundance of the two products was determined by a combination of chromatography and electrospray, ESI, mass spectrometry.^18^ This study revealed a positive reaction constant (‘ρ’) of 1.8, which indicated that the electron density distribution on the carbon atom of the carbonyl group in the benzil increased substantially during the rate-limiting step. In turn, this discovery confirmed that the benzil behaves as an electrophile in the condensation, as might be expected intuitively. This protocol was successfully extended to the complementary process in which phenylenediamine competes with either a mono- or di-substituted phenylenediamine to condense with benzil to form a mono- or di-substituted diphenylquinoxaline.^19^ A negative ρ value of −0.96, which indicates that there is an appreciable reduction in the electron density on the amino group(s) during the rate-limiting step, established that the phenylenediamine acts as a nucleophile in the condensation. Moreover, analysis of the mixture of products formed by transmission through a nebuliser was found to be conveniently and reliably achieved by gas chromatography-mass spectrometry (GCMS).

These condensations in microdroplets occur under conditions in which the reactants are at least at a good approximation to equilibrium. As such, the protocol involving competition experiments appears to be sufficiently valid to allow useful structure reactivity relationships to be devised in these accelerated reactions that effectively occur in solution. Competition experiments can be exploited in the gas-phase dissociation of isolated ions in mass spectrometers to obtain valuable mechanistic information. However, caution is needed because several factors^9,10^ must be taken into account when considering the influence of a substituent on the rate of fragmentation, which may involve ions of very different internal energies. Two classical means of overcoming these problems are to reduce the energy of the ionising electrons in electron ionisation (EI) mass spectrometry, thereby minimising complications caused by the possibility of secondary fragmentations, or to study the reactions of metastable ions, which dissociate with a relatively well-defined and small range of excess energy in the transition state.^20,21^ Thus, the competition between elimination of either C_n_H_2n_ or C_m_H_2m_ from metastable immonium ions of general structure [C_n+1_H_2n+3_(C_m+1_H_2m+3_)N=CH_2_]^+^ gave valuable mechanistic information by revealing a strong preference for an initial hydrogen transfer from the more heavily substituted γ-carbon atom.^22–27^

**Scheme 1.**
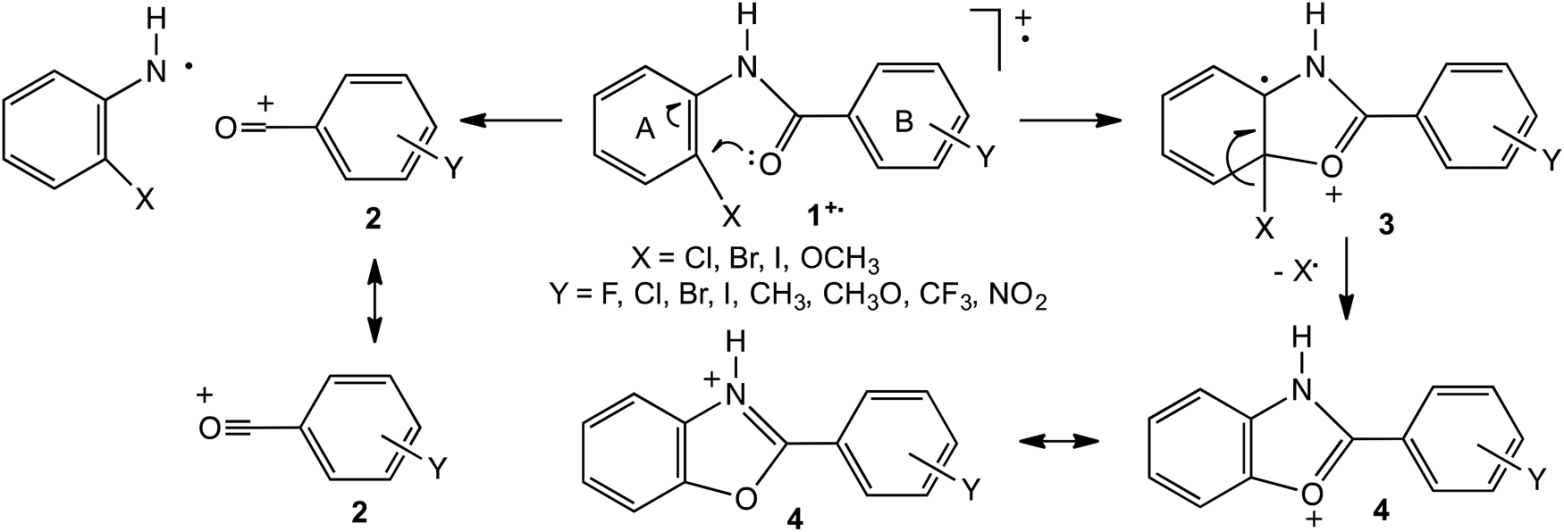
Principal dissociation routes of ionised substituted benzanilides.

The scope of the method based on the proportions of alternative processes was expanded by investigating the *intramolecular* competition in the fragmentation of four series of ionised 2-substituted benzanilides, 2-XC_6_H_4_NHCOC_6_H_4_Y**^+^** ˙, (X=CH_3_O, Cl, Br, I; Y=H, F, Cl, Br, I, CH_3_, OCH_3_, CF_3_, NO_2_).^28^ Two principal fragmentations are observed. Firstly, direct cleavage of the amide linkage in **1^+^˙** gives a substituted benzoyl cation, **2**, containing ring B. Secondly, a rearrangement known as a ‘proximity effect’ occurs involving cyclisation to **3** that ultimately results in loss of the original 2-substituent in ring A to give a very stable protonated benzoxazole, **4**, Scheme 1.

Good Hammett plots were generated from the relative intensities, RIs, of the signals corresponding to the two fragment ions, **2** and **4**. An improved correlation was obtained by using σ^+^ and σ^−^ constants for certain substituents (OCH_3_ and NO_2_, respectively) in which conjugation with the reaction site is highly effective. This refinement gave R^2^ correlation values between 0.90 and 0.96; the derived ρ constant declined systematically on descending the series (OCH_3_=0.56, Cl=0.54, Br=0.52, I=0.38), thus giving further insight into the mechanism of the proximity effect.^28^

**Scheme 2.**
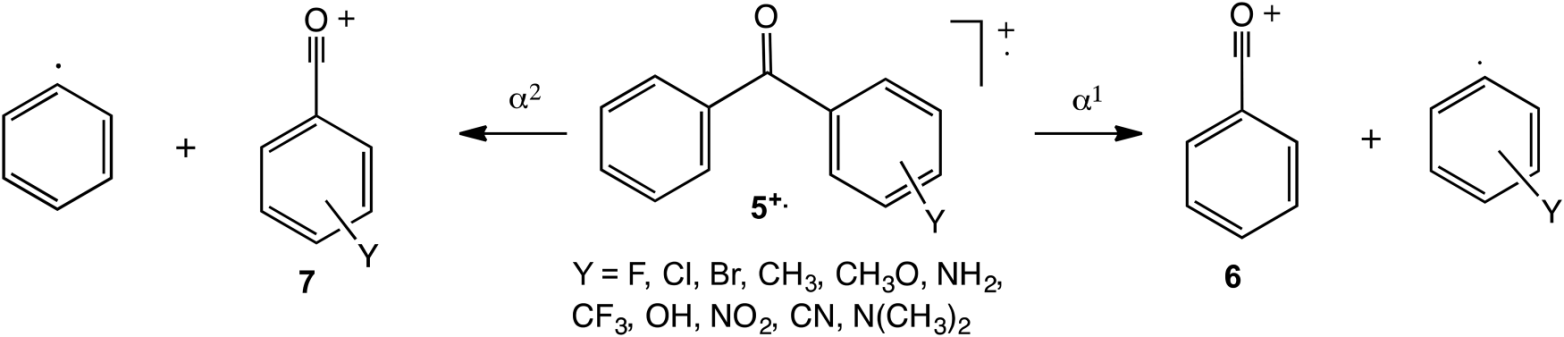
Principal dissociation routes of ionised substituted benzophenones.

Substituted ionised benzophenones, **5^+^˙**, undergo two competing simple cleavages, α^1^ or α^2^, to form, respectively, either the benzoyl cation, **6**, and a substituted phenyl radical, or a substituted benzoyl cation, **7**, and the phenyl radical, Scheme 2. Construction of a Hammett plot based on the RIs of the signals corresponding to **6** and **7** permits a comparison of the method based on competing reactions and that previously applied^8–11^ on the basis of the relative abundances, RAs, of parent and fragment ions. In addition, it raises the possibility of extending this newer methodology to probe other systems, including the fragmentation of ionised substituted dibenzylideneacetones, YC_6_H_4_CH=CHCOCH=CHC_6_H_5_.

## Experimental

### Synthesis

Almost all the substituted benzophenones were available commercially. The few that were not were prepared by addition of the requisite substituted benzaldehyde, YC_6_H_4_CHO, to a slight excess (1.2 equivalents) of phenyl magnesium bromide in diethyl ether followed by work-up with saturated aqueous ammonium chloride solution and extraction of the resultant substituted benzhydrols into dichloromethane. Oxidation with aqueous sodium dichromate solution^29^ then gave the desired substituted benzophenone, which was purified by recrystallisation or distillation at reduced pressure.

The substituted dibenzylideneacetones were obtained from freshly prepared and recrystallised benzylideneacetone^30^ by a refinement of a published procedure.^31^ Equimolar quantities (typically 10 mmol) of benzylideneacetone and the requisite benzaldehyde, YC_6_H_4_CH=O, were dissolved in the minimum amount of methanol (typically 5–10 mL) and sufficient aqueous potassium hydroxide solution (10% w/v, typically 7–9 drops) was added to the magnetically stirred solution to bring the pH to 9. A yellow colouration developed almost immediately, usually followed shortly afterwards by the precipitation of the desired product as a lemon-yellow solid. Stirring was continued overnight, whereupon the solid product was isolated by filtration in a Büchner funnel, washed with water until free from base (to pH 6–7) and recrystallised from either ethanol/water or ethanol. Yields varied from 40 to 75%.

No impurities in any of the products prepared in the laboratory were detected by ^1^H NMR or mass spectrometry.

### Mass spectrometry

Separate solutions of the samples in methanol or dichloromethane, as appropriate, at a concentration of 0.067 mol L^−1^ were prepared and admitted to a 7890 gas chromatograph attached to a 5975 EI Inert MSD (Agilent Technologies, USA). Gas chromatography, GC, was achieved with a 30 m × 0.25 mm 5% diphenyl low-polarity fused-silica capillary column, using helium or hydrogen as the carrier gas at a flow rate of 1.2 mL min^−1^, with a 50:1 split. Ionisation was effected with electrons having a nominal energy of 20, 30, 50, and 70 eV in separate series of experiments. The temperature of the source and quadrupole was 230 and 150 °C, respectively. The initial temperature of the GC was 50 °C, increasing linearly at 25 °C min^−1^ to 350 °C, where it was maintained for 2 min. Data were acquired over the m/z range 50–600. Quoted ratios are the averages of three independent runs.

The possibility of errors in the ratio of RAs of fragment ions because of spectral skewing^32^ across the GC peaks was considered to be remote because data were extracted manually from the same ‘early’ part of the peak before any potential problems associated with saturation of the detector could arise. Distortion of data caused by spectral skewing can become problematic when the GC peak is so narrow that only a very small number of scans are possible. The instrumental conditions that applied during the acquisition of the data reported in this paper permitted 30–50 scans for each GC peak. Moreover, the data reported in this work were the means of three independent GCMS runs; the standard deviations in the ratios indicate that the results are reproducible, which is most unlikely to have occurred if there had been distortion owing to spectral skewing. In addition, careful comparison of the spectra obtained in each individual scan revealed no significant variations in the reported ratios.

## Results and discussion

 Table 1 summarises the ratios of the RAs of [C_6_H_5_CO]^+^, **6**, and [YC_6_H_4_CO]^+^, **7**, formed by loss of YC_6_H_4_˙ and C_6_H_5_˙, respectively, by α^1^ and α^2^ cleavage of **5^+^˙**.

**Table 1.** Ratio of relative abundance^a^ of [C_6_H_5_CO]^+^ and [YC_6_H_4_CO]^+^ formed from YC_6_H_4_COC_6_H_5_^+^.

Y^b^	[C_6_H_5_CO]^+^/[YC_6_H_4_CO]^+^	Log_10_([C_6_H_5_CO]^+^/[YC_6_H_4_CO]^+^)	SD^c^
4NO_2_	6.56	0.82	0.095
3Br	5.26	0.72	0.169
4CN	3.73	0.57	0.010
3Cl	3.40	0.53	0.066
4CF_3_	2.89	0.46	0.031
3CF_3_	2.72	0.43	0.087
3F	2.69	0.43	0.117
4Br	1.79	0.25	0.007
3OH	1.37	0.14	0.006
4Cl	1.16	0.06	0.005
H	[1^d^]	0	N/A
3OCH_3_	0.89	−0.05	0.004
4F	0.73	−0.14	0.001
4CH_3_	0.28	−0.55	0.002
4OH	0.19	−0.71	0.003
4N(CH_3_)_2_	0.12	−0.90	0.001
4OCH_3_	0.12	−0.93	0.003
4NH_2_	0.06	−1.24	0.001

a Relative abundance measured by the peak height of the corresponding signal in the 70 eV electron ionisation spectrum.

b The number in this column defines the position of the substituent, Y. The substituents are arranged from top to bottom in order of decreasing [C_6_H_5_CO]^+^/[YC_6_H_4_CO]^+^ ratio. This order corresponds approximately to descending electron-withdrawing power (or increasing electron-donating power).

c Standard deviation, quoted in log_10_ units.

d Only one signal for [C_6_H_5_CO]^+^ and [YC_6_H_4_CO]^+^ is observed in the symmetrical case with Y=H.

**Figure 1.**
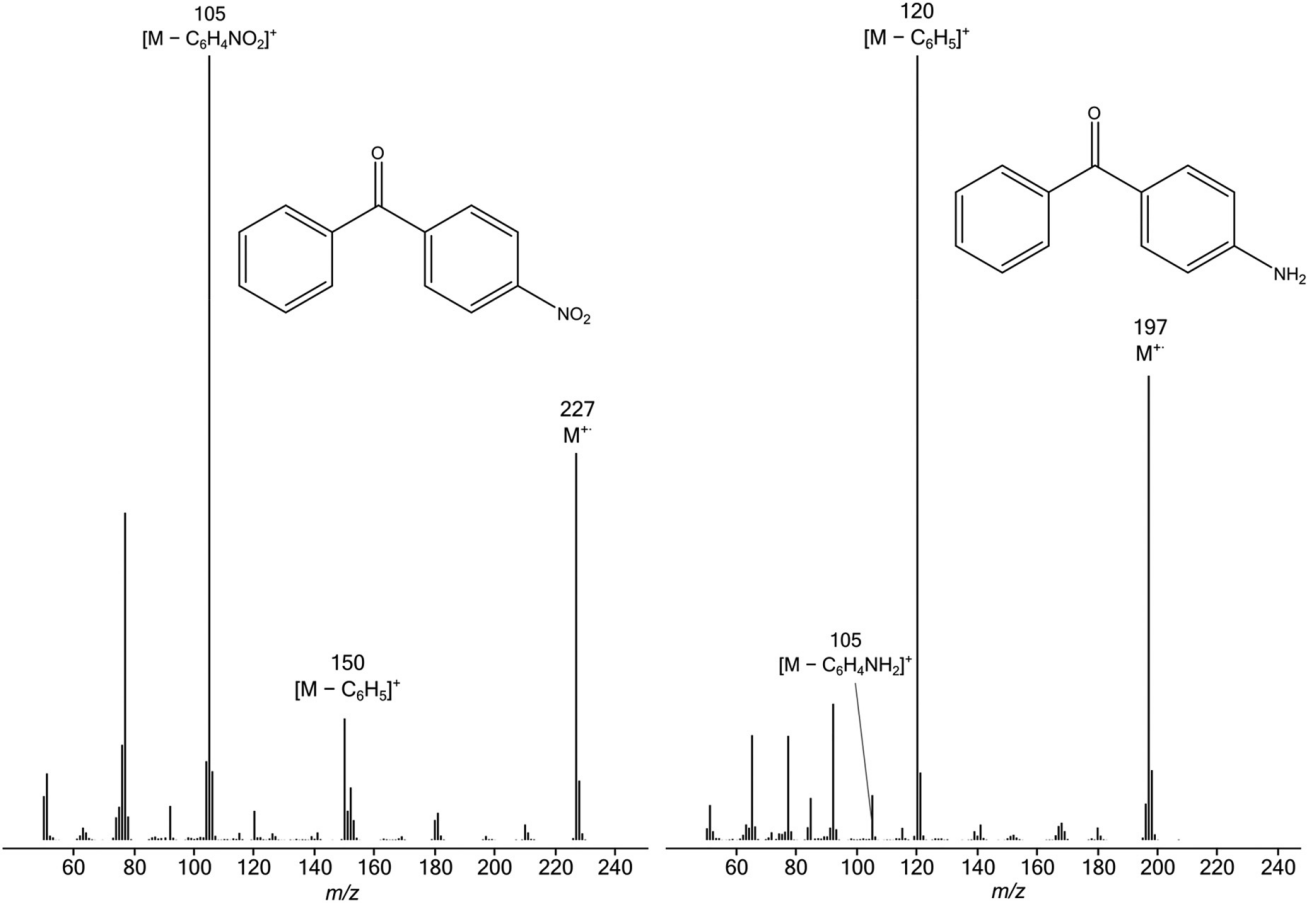
EI mass spectra of 4-O_2_NC_6_H_4_COC_6_H_5_ (left) and 4-H_2_NC_6_H_4_COC_6_H_5_ (right).

An important general trend is evident in the data of Table 1: more electron-withdrawing substituents favour the formation of [C_6_H_5_CO]^+^ rather than [YC_6_H_4_CO]^+^, as would be expected intuitively because these substituents destabilise the substituted benzoyl cation, but have relatively little effect on the substituted phenyl radical. Conversely, electron-donating groups increase the electron density in the aromatic ring, thus stabilising the substituted benzoyl cation and favouring its formation in preference to production of the parent benzoyl cation. These trends are illustrated by the representative EI spectra shown in Figure 1. In the spectrum of 4-H_2_NC_6_H_4_COC_6_H_5_, formation of [M-C_6_H_5_]^+^ is favoured because the 4-amino substituent stabilises the resultant [H_2_NC_6_H_4_CO]^+^ fragment ion by the + M mesomeric effect. Conversely, in the spectrum of 4-O_2_NC_6_H_4_COC_6_H_5_, formation of [M-O_2_NC_6_H_4_]^+^ is favoured because the 4-nitro substituent destabilises the resultant [O_2_NC_6_H_4_CO]^+^ fragment ion by the −M mesomeric effect, thus leading to preferential formation of the more stable unsubstituted benzoyl cation, [C_6_H_5_CO]^+^.

**Figure 2.**
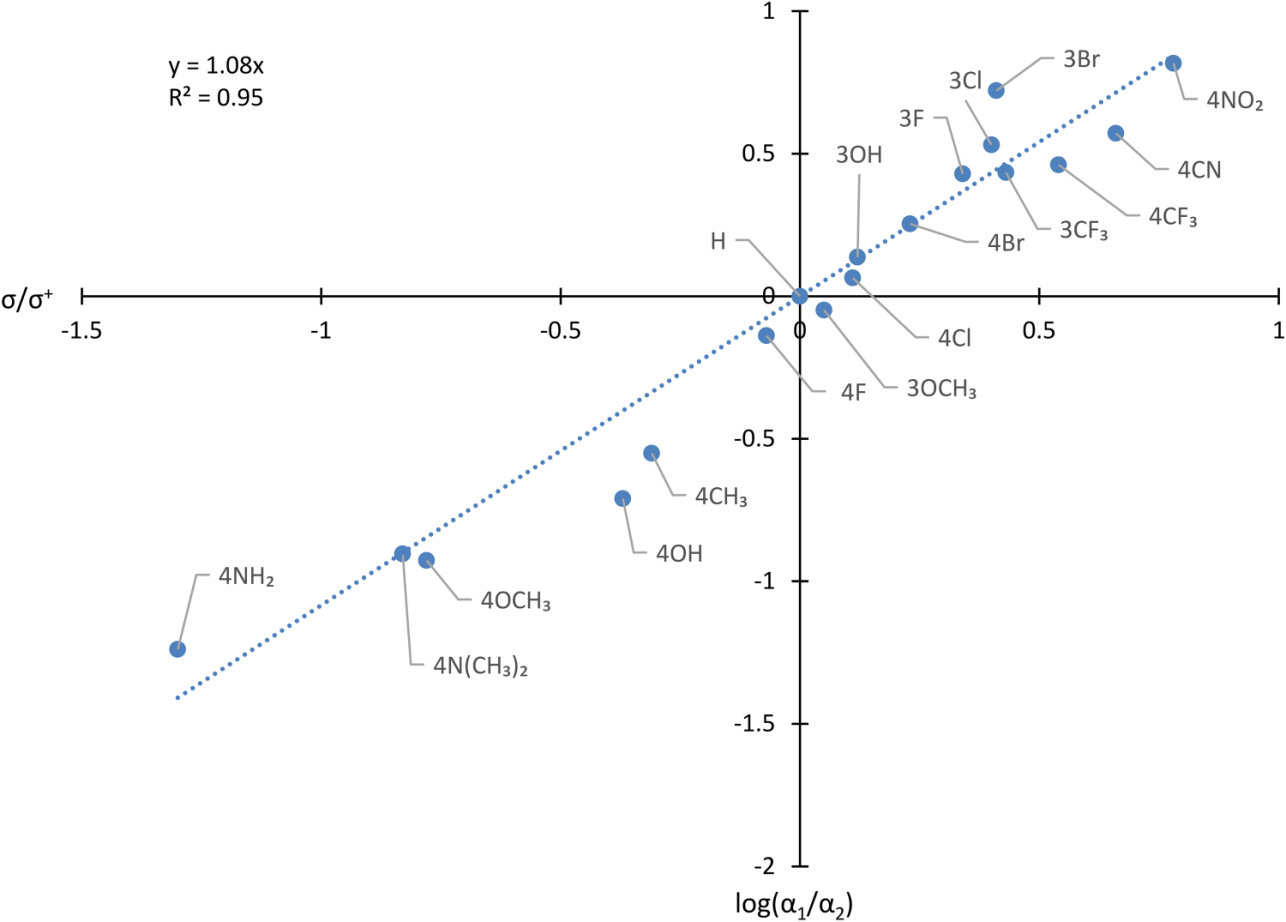
Hammett plot showing the influence of Y on the ratio of relative abundance of [C_6_H_5_CO]^+^/[YC_6_H_4_CO]^+^ in the spectra of YC_6_H_4_COC_6_H_5_.

This qualitative trend can be interpreted in greater detail by constructing the Hammett plot of Figure 2. As previously described,^28^ σ^+^ constants were employed in cases [Y=CH_3_O and NH_2_ in the 4-position] where the substituent interacts strongly with the aromatic ring by a positive (+M) mesomeric effect.

Three deductions can be made from Figure 2.

Firstly, there is a good correlation, with an R^2^ value of 0.95. This finding shows that the approach based on competition experiments can produce useful data of high quality. The small standard deviations (average 0.036 log_10_ units) in the data confirm this point.

Secondly, the derived Hammett reaction constant, ρ, of 1.08 agrees well with the value of 1.01 previously obtained by analysis of the RAs of parent and fragment ions.^8^ This good agreement indicates that the new method can give similar data and insight to the older procedure. Moreover, this new method potentially has practical advantages. It may be easily employed in analysing a series of compounds that are amenable to automated analysis by GCMS. In addition, it might be applicable in systems for which the signal for the molecular ion is weak (thus introducing errors arising from uncertainties associated with small numbers) or even absent. Comparisons between more than two fragmentations would, at least in principle, be feasible. The excellent agreement between the ρ value found in this work and that reported previously is strong evidence that construction of a Hammett correlation based on the RAs from the mass spectra recorded by GCMS offers a reliable alternative to earlier methods. In addition, this agreement confirms that spectral skewing does not pose a problem provided that the data are extracted manually from the same early part of a relatively broad GC peak which may be scanned many times to acquire reliable data. Furthermore, despite the well-known wide range of the energies of dissociating ions formed by EI,^21,33–35^ an approach based on the RAs of ions formed in 70 eV spectra can be applied, at least in some cases.

Thirdly, the positive ρ value of 1.08 confirms that an electron-withdrawing substituent increases the rate of production of [C_6_H_5_CO]^+^ relative to that for formation of [YC_6_H_4_CO]^+^ from the gaseous molecular ion [YC_6_H_4_COC_6_H_5_]^+^˙. However, the Hammett correlation gives at least a semi-quantitative description of the fragmentation, thus bringing this important simple cleavage within the compass of physical organic chemistry.

One potential drawback of the method is that it neglects the effect of secondary fragmentation of [C_6_H_5_CO]^+^ and [YC_6_H_4_CO]^+^, which might reduce the RA of one of these two primary fragment ions more than the other. In order to address this concern, the work was repeated under the same conditions at various ionising electron energies (50, 30, and 20 eV). Reducing the energy of the ionising electrons, which should decrease the tendency of the primary (and secondary) fragment ions to dissociate,^36–38^ had little effect on ρ, which varied only slightly in the range 0.99–1.08, and even less on R^2^, which remained almost constant at 0.95–0.96. Various approaches to making allowance for the secondary fragment ions, [C_6_H_5_]^+^ and [C_6_H_4_X]^+^, formed by expulsion of CO from [C_6_H_5_CO]^+^ and [YC_6_H_4_CO]^+^, proved to be counterproductive: R^2^ fell to between 0.50 and 0.82. These additional experiments suggest strongly that the approach to constructing Hammett correlations based on the relative abundance of primary fragment ions formed with ionisation with 70 eV electrons works well in at least some systems without complicating the analysis by considering secondary fragmentations. There are significant disadvantages in employing lower ionising electron energies: fewer ions are produced, thus decreasing signal intensity and increasing errors; furthermore, there is a greater risk of filament damage, especially when the GCMS is operated automatically.

**Scheme 3.**
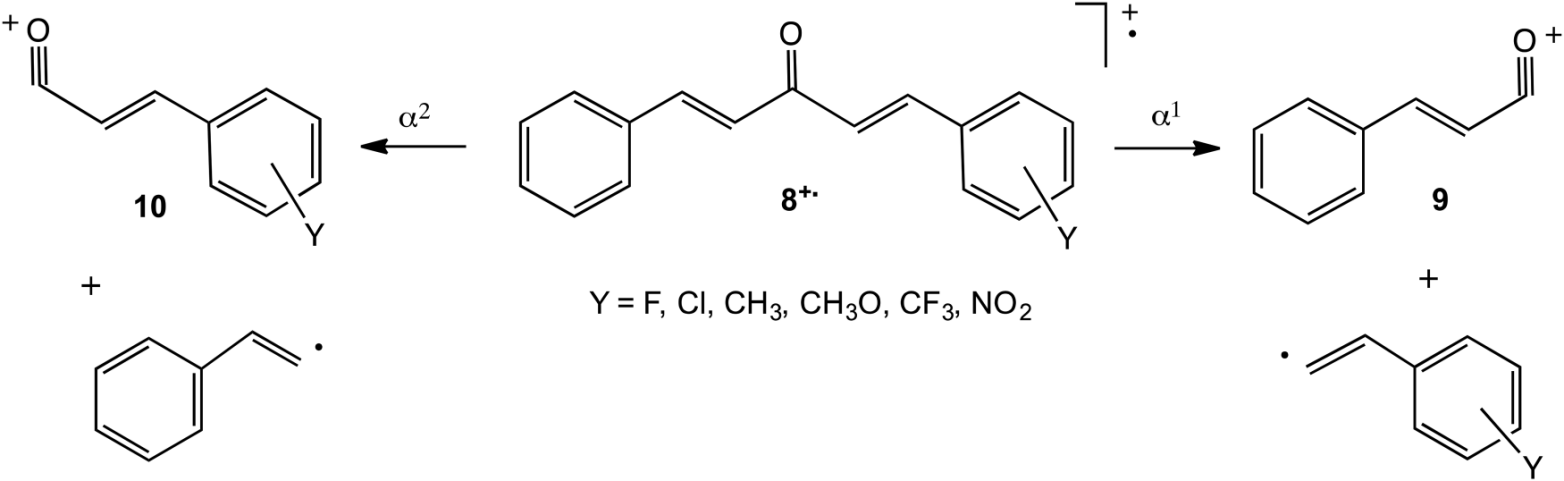
Relevant dissociation routes of ionised substituted dibenzylideneacetones.

In order to investigate whether this approach could be extended to competing simple cleavages of other ionised carbonyl compounds, the 70 eV EI spectra of a series of substituted dibenzylideneacetones YC_6_H_4_CH=CHCOCH=CHC_6_H_5_, **8**, were recorded. Fission of the C-C bond on either side of the carbonyl group in **8^+.^** leads either to the cinnamoyl cation, [C_6_H_5_CH=CHCO]^+^, **9**, and a substituted 2-phenylvinyl radical, YC_6_H_4_CH=CH˙, or to the substituted cinnamoyl cation, [YC_6_H_4_CH=CHCO]^+^, **10**, and the 2-phenylvinyl radical, C_6_H_5_CH=CH˙, Scheme 3. These two processes, α^1^ and α^2^, respectively, are analogous to those for the corresponding ionised substituted benzophenones.

**Figure 3.**
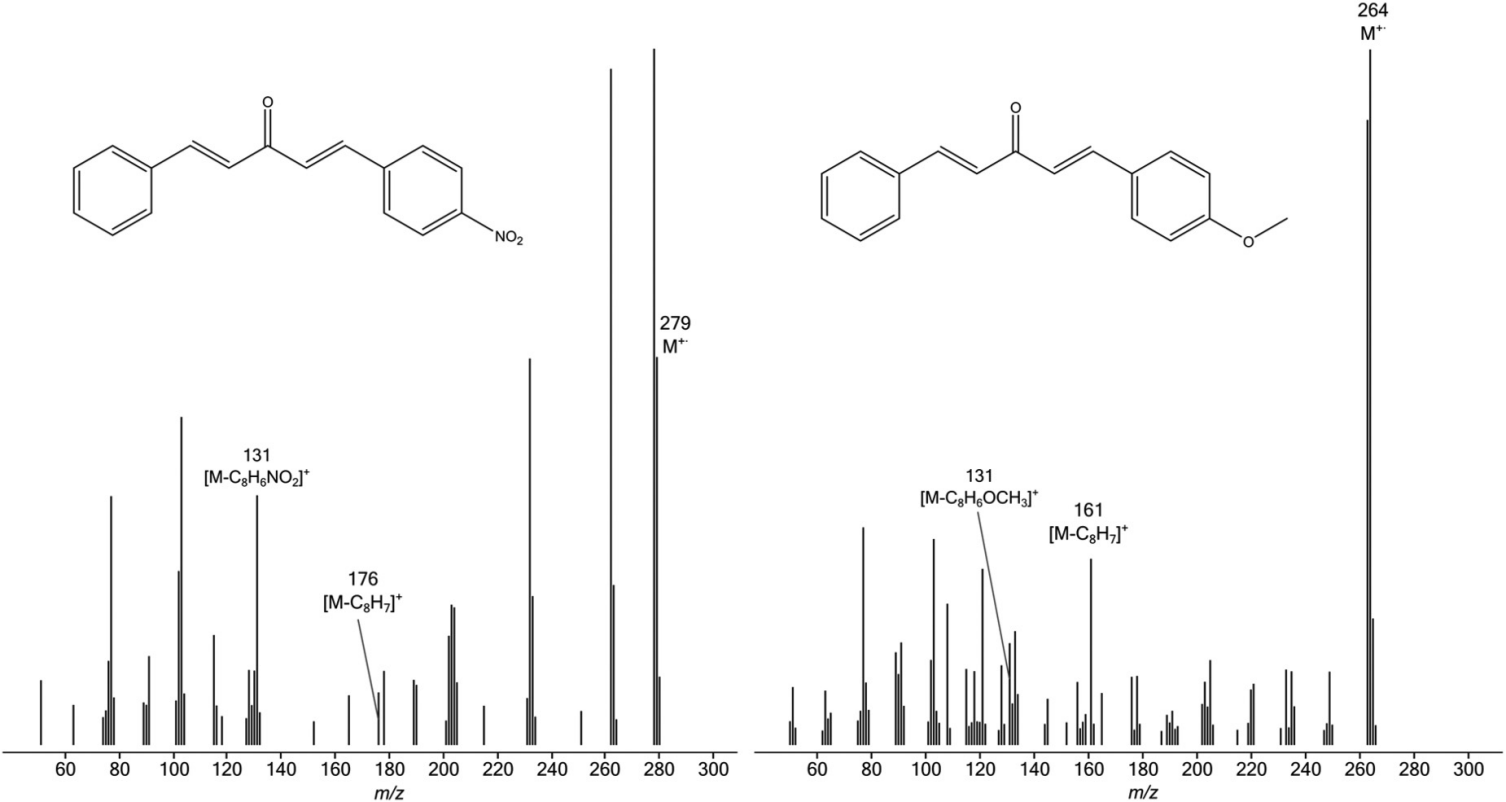
EI mass spectra of 4-O_2_NC_6_H_4_CH=CHCOCH=CHC_6_H_5_ (left) and 4-CH_3_OC_6_H_4_CH=CHCOCH=CHC_6_H_5_ (right).

In contrast to the spectra of **5**, which are dominated by signals corresponding to simple cleavage to form **6** and **7**, the spectra of **8** contain peaks attributable to ions other than **9** and **10**. Representative spectra are shown in Figure 3.

**Figure 4.**
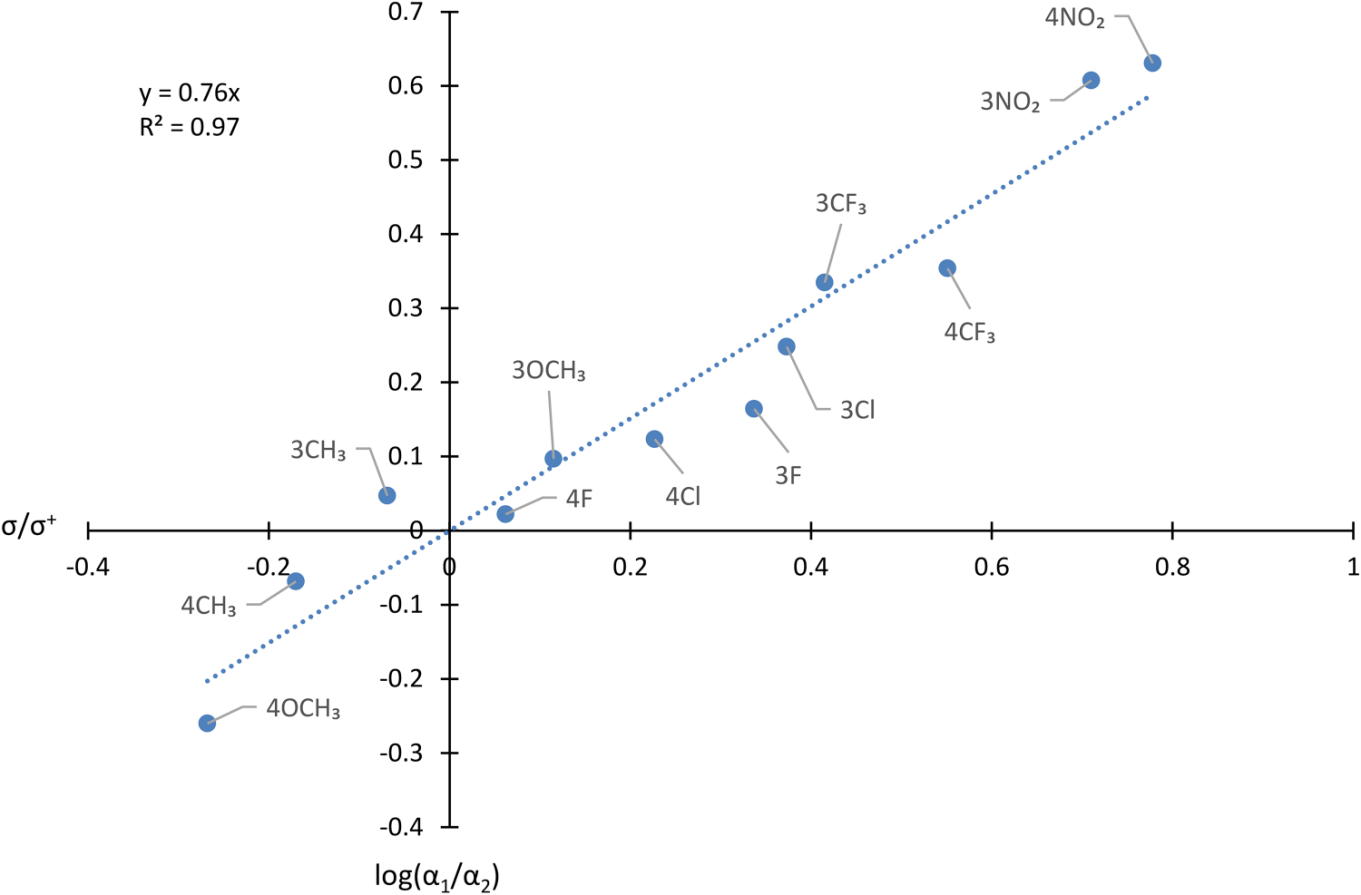
Hammett plot showing the influence of Y on the ratio of relative abundance of [C_6_H_5_CH=CHCO]^+^/[YC_6_H_4_CH=CHCO]^+^ in the spectra of YC_6_H_4_CH=CHCOCH=CHC_6_H_5_.

Despite the complications caused by other fragmentations, the RAs of **9** and **10** are sufficiently strong to permit the compilation of the data of Table 2, from which the Hammett plot shown in Figure 4 is derived.

**Table 2.** Ratio of relative abundance^a^ of [C_6_H_5_CH=CHCO]^+^ and [YC_6_H_4_CH=CHCO]^+^ formed from YC_6_H_4_CH=CHCOCH=CHC_6_H_5_^+^.

Y^b^	[C_6_H_5_CH=CHCO]^+^/[YC_6_H_4_CH=CHCO]^+^	Log_10_([C_6_H_5_CH=CHCO]^+^/[YC_6_H_4_CH=CHCO]^+^)	SD^c^
4NO_2_	4.27	0.63	0.027
3NO_2_	4.05	0.61	0.006
4CF_3_	2.26	0.35	0.082
3CF_3_	2.16	0.33	0.017
3Cl	1.77	0.25	0.059
3F	1.46	0.16	0.017
3OCH_3_	1.25	0.10	0.009
3CH_3_	1.11	0.05	0.010
4F	1.05	0.02	0.039
H	[1^d^]	0	N/A
4CH_3_	0.85	−0.07	0.082
4OCH_3_	0.55	−0.26	0.050

a Relative abundance measured by the peak height of the corresponding signal in the 70 eV electron ionisation spectrum.

b The number in this column defines the position of the substituent, Y. The substituents are arranged from top to bottom in order of decreasing [C_6_H_5_CH=CHCO]^+^/[YC_6_H_4_CH=CHCO]^+^ ratio. This order corresponds approximately to descending electron-withdrawing power (or increasing electron-donating power).

c Standard deviation, quoted in log_10_ units.

d Only one signal for [C_6_H_5_CH=CHCO]^+^ and [YC_6_H_4_CH=CHCO]^+^ is observed in the symmetrical case with Y=H.

The following deductions may be made from Table 2 and Figure 4.

Firstly, as was the case for **5^+^˙**, the substituent Y exercises an influence on the stability of the primary fragment ion [YC_6_H_4_CH=CHCO]^+^ that may be interpreted in a qualitative manner. When Y is electron-withdrawing, [YC_6_H_4_CH=CHCO]^+^ is destabilised by Y, relative to [C_6_H_5_CH=CHCO]^+^, thus favouring formation of **9**, as is observed in the spectrum of 4-O_2_NC_6_H_4_CH=CHCOCH=CHC_6_H_5_, because [O_2_NC_6_H_4_CH=CHCO]^+^ is less stable than [C_6_H_5_CH=CHCO]^+^. Conversely, when Y is electron-donating, [YC_6_H_4_CH=CHCO]^+^ is stabilised by Y, relative to [C_6_H_5_CH=CHCO]^+^, and **10** is formed preferentially, as is seen in the spectrum of 4-CH_3_OC_6_H_4_CH=CHCOCH=CHC_6_H_5_, since [CH_3_OC_6_H_4_CH=CHCO]^+^ is more stable than [C_6_H_5_CH=CHCO]^+^. The effect of the substituent on the stability of the arylvinyl radical is far smaller and can be neglected to a first approximation.

Secondly, the Hammett plot shown in Figure 2 shows that this qualitative correlation, which might have been anticipated intuitively, may be placed on a more quantitative basis. The ρ constant of 0.76 indicates that the electron density decreases at the carbon atom of the carbonyl group during the dissociation. The R^2^ value of 0.97 reveals that the correlation is good.

Thirdly, the value of ρ of 0.76 derived from competition in the dissociation of YC_6_H_4_CH=CHCOCH=CHC_6_H_5_^+^˙ is appreciably and reproducibly lower than that of 1.08 found for the corresponding fragmentation of YC_6_H_4_COC_6_H_5_^+^˙. This finding indicates that the influence of Y on the stability of [YC_6_H_4_CH=CHCO]^+^, though significant, is lower than on the stability of [YC_6_H_4_CO]^+^. In turn, these results show that the effect of conjugation in [YC_6_H_4_CH=CHCO]^+^ remains strong, but is diminished relative to that in [YC_6_H_4_CO]^+^. In other words, the π-conjugation, which connects the aromatic ring and the carbonyl group in the ‘vinylogous’ cation, [YC_6_H_4_CH=CHCO]^+^, is highly effective, but not quite as good as when the aromatic ring is directly bound to the cationic site.

Fourthly, the success of the approach based on competition experiments in this system indicates that it has potential to probe the mechanism of other fragmentations. However, certain caveats must be applied. The most important is that the dissociation routes under consideration must occur sufficiently rapidly to give fragment ions with reliable and significant RAs. In other words, the occurrence of alternative processes may mean that the approach is impractical. In the present case, it is necessary to exclude substituents that open up new possibilities for fragmentation that occur so rapidly that the desired simple cleavage cannot be studied effectively. Thus, when Y=Br or I, strong [M-Br]^+^ and [M-I]^+^ signals, respectively, appear in the spectra that pre-empt to a considerable degree the formation of [BrC_6_H_4_CH=CHCO]^+^ and [IC_6_H_4_CH=CHCO]^+^. Similarly, attempts to devise a Hammett correlation to show the effect of Y or X in influencing the dissociation by simple cleavage of ionised substituted chalcones of general structure YC_6_H_4_CH=CHCOC_6_H_5_ (to form [YC_6_H_4_CH=CHCO]^+^ or [C_6_H_5_CO]^+^) and C_6_H_5_CH=CHCOC_6_H_4_X (to form [C_6_H_5_CH=CHCO]^+^ or [XC_6_H_4_CO]^+^) were not so successful. It appears that the approach works best when the competition between two closely comparable fragmentations is investigated.

## Conclusion

Comparison of the relative abundances of ions formed in competing fragmentations of molecular ions offers an alternative means of constructing Hammett correlations, thus furnishing valuable information about the mechanisms of these processes. Application of this method in the case of ionised substituted benzophenones gives a good correlation (R^2^=0.95) with a closely similar reaction constant (1.08) to that (1.01) found in earlier work.^8^ Extension of the approach to the dissociation of ionised substituted dibenzylideneacetones reveals another good correlation (R^2^=0.97) with a somewhat lower reaction constant of 0.76, provided that certain substituents (Br and I) are excluded in which other processes specifically associated with the substituent dominate the fragmentation. The positive reaction constants for simple cleavage of both ionised substituted benzophenones and dibenzylideneacetones indicates that the electron density on the carbon atom of the carbonyl group deceases (or that positive charge on that atom increases) during the formation of either the benzoyl or cinnamoyl cation. The smaller reaction constant in the dissociation to form the cinnamoyl cation reveals that the influence of the substituent persists through extended π-conjugation, but the stabilisation or destabilisation is slightly less pronounced than when the aromatic ring is directly bound to the cationic site.

## Supplemental Material

sj-docx-1-ems-10.1177_14690667231184363 - Supplemental material for Hammett correlation in competition experiments in dissociation of ionised substituted benzophenones and dibenzylideneacetones Click here for additional data file. Supplemental material, sj-docx-1-ems-10.1177_14690667231184363 for Hammett correlation in competition experiments in dissociation of ionised substituted benzophenones and dibenzylideneacetones by Nathan W Fenwick, Richard Telford, William H C Martin and Richard D Bowen in European Journal of Mass Spectrometry
