# Supplementary material for: Hammett correlation in competition experiments in dissociation of ionised substituted benzophenones and dibenzylideneacetones
Source: Eur J Mass Spectrom (Chichester). 2023 Jul 5;29(4):211–9. doi: 10.1177/14690667231184363 (PMC10466955; doi:10.1177/14690667231184363)
Supplement: sj-docx-1-ems-10.1177_14690667231184363 - Supplemental material for Hammett correlation in competition experiments in dissociation of ionised substituted benzophenones and dibenzylideneacetones [file sj-docx-1-ems-10.1177_14690667231184363.docx]

Supplemental Information:

Spectra shown are the ensemble spectra for the GCMS peaks for each compound analysed, recorded using the method described in the body of the paper.

Representative EIMS Spectra:

3-F-benzophenone:

Structure:

M^+•^ (C_13_H_9_FO): 200 amu

4-F-benzophenone:

Structure:

M^+•^ (C_13_H_9_FO): 200

3-Cl-benzophenone:

Structure:

M^+•^ (C_13_H_9_ClO): 216

4-Cl-benzophenone:

Structure:

M^+•^ (C_13_H_9_ClO): 216

3-Br-benzophenone:

Structure:

M^+•^ (C_13_H_9_BrO): 260

4-Br-benzophenone:

Structure:

M^+•^ (C_13_H_9_BrO): 260

4-CH_3_-benzophenone:

Structure:

M^+•^ (C_14_H_12_O): 196

3-CH_3_O-benzophenone:

Structure:

M^+•^ (C_14_H_12_O_2_): 212

4-CH_3_O-benzophenone:

Structure:

M^+•^ (C_14_H_12_O_2_): 212

4-NH_2_-benzophenone:

Structure:

M^+•^ (C_13_H_11_NO): 197

3-CF_3_-benzophenone:

Structure:

M^+•^ (C_14_H_9_F_3_O): 250

4-CF_3_-benzophenone:

Structure:

M^+•^ (C_14_H_9_F_3_O): 250

4-N(CH_3_)_2_-benzophenone:

Structure:

M^+•^ (C_15_H_15_NO): 225

3-OH-benzophenone:

Structure:

M^+•^ (C_13_H_10_O_2_): 198

4-OH-benzophenone:

Structure:

M^+•^ (C_13_H_10_O_2_): 198

4-NO_2_-benzophenone:

Structure:

M^+•^ (C_13_H_9_NO_3_): 227

4-CN-benzophenone:

Structure:

M^+•^ (C_14_H_9_NO): 207

3-F-dibenzylideneacetone:

Structure:

M^+•^ (C_17_H_13_FO): 252

4-F-dibenzylideneacetone:

Structure:

M^+•^ (C_17_H_13_FO): 252

3-Cl-dibenzylideneacetone:

Structure:

M^+•^ (C_17_H_13_ClO): 268

4-Cl-dibenzylideneacetone:

Structure:

M^+•^ (C_17_H_13_ClO): 268

3-CH_3_-dibenzylideneacetone:

Structure:

M^+•^ (C_18_H_16_O): 248

4-CH_3_-dibenzylideneacetone:

Structure:

M^+•^ (C_18_H_16_O): 248

3-OCH_3_-dibenzylideneacetone:

Structure:

M^+•^ (C_18_H_16_O_2_): 264

4-OCH_3_-dibenzylideneacetone:

Structure:

M^+•^ (C_18_H_16_O_2_): 264

3-CF_3_-dibenzylideneacetone:

Structure:

M^+•^ (C_18_H_13_F_3_O): 302

4-CF_3_-dibenzylideneacetone:

Structure:

M^+•^ (C_18_H_13_F_3_O): 302

3-NO_2_-dibenzylideneacetone:

Structure:

M^+•^ (C_17_H_13_NO_3_): 279

4-NO_2_-dibenzylideneacetone:

Structure:

M^+•^ (C_17_H_13_NO_3_): 279
